# Supplementary material for: Time-series transcriptome analysis identified differentially expressed genes in broiler chicken infected with mixed Eimeria species
Source: Front Genet. 2022 Aug 8;13:886781. doi: 10.3389/fgene.2022.886781 (PMC9393255; doi:10.3389/fgene.2022.886781)
Supplement: Supplementary file 2 [file DataSheet1.ZIP › 7dpi_GO.Gsea.1625106495923/GOBP_PHAGOSOME_ACIDIFICATION.html]

Details for gene set GOBP\_PHAGOSOME\_ACIDIFICATION[GSEA]

|  || Dataset | TMM\_7dpi\_gct\_format\_7dpi\_gct\_format.Class\_4dpi.cls #PC\_versus\_NC.Class\_4dpi.cls #PC\_versus\_NC\_repos |
| Phenotype | Class\_4dpi.cls#PC\_versus\_NC\_repos |
| Upregulated in class | 1 |
| GeneSet | GOBP\_PHAGOSOME\_ACIDIFICATION |
| Enrichment Score (ES) | 0.8602565 |
| Normalized Enrichment Score (NES) | 3.0681756 |
| Nominal p-value | 0.0 |
| FDR q-value | 0.0 |
| FWER p-Value | 0.0 |
Table: GSEA Results Summary

  

Fig 1: Enrichment plot: GOBP\_PHAGOSOME\_ACIDIFICATION      
 Profile of the Running ES Score & Positions of GeneSet Members on the Rank Ordered List

  

| SYMBOL | TITLE | RANK IN GENE LIST | RANK METRIC SCORE | RUNNING ES | CORE ENRICHMENT || 1 | ATP6V1C2 | na | 4 | 2.128 | 0.1222 | Yes |
| 2 | ATP6V0A4 | na | 19 | 1.686 | 0.2181 | Yes |
| 3 | ATP6V0D2 | na | 24 | 1.636 | 0.3120 | Yes |
| 4 | ATP6V0C | na | 30 | 1.603 | 0.4039 | Yes |
| 5 | ATP6V1D | na | 46 | 1.458 | 0.4866 | Yes |
| 6 | ATP6V1E1 | na | 61 | 1.395 | 0.5658 | Yes |
| 7 | ATP6V0E1 | na | 72 | 1.354 | 0.6430 | Yes |
| 8 | ATP6V1H | na | 80 | 1.313 | 0.7180 | Yes |
| 9 | ATP6V0B | na | 100 | 1.252 | 0.7885 | Yes |
| 10 | ATP6V1G3 | na | 102 | 1.247 | 0.8603 | Yes |
| 11 | SLAMF8 | na | 982 | 0.529 | 0.8174 | No |
| 12 | ATP6V1G1 | na | 2444 | 0.199 | 0.7070 | No |
| 13 | RAB7A | na | 3623 | 0.051 | 0.6117 | No |
| 14 | TCIRG1 | na | 3866 | 0.020 | 0.5926 | No |
| 15 | ATP6V1C1 | na | 4112 | -0.007 | 0.5726 | No |
| 16 | ATP6V0A1 | na | 4522 | -0.052 | 0.5415 | No |
| 17 | ATP6V0D1 | na | 5522 | -0.151 | 0.4669 | No |
| 18 | ATP6V1A | na | 5917 | -0.192 | 0.4451 | No |
| 19 | ATP6V1B2 | na | 5973 | -0.199 | 0.4520 | No |
| 20 | ATP6V0E2 | na | 6836 | -0.290 | 0.3968 | No |
| 21 | ATP6V0A2 | na | 9318 | -0.602 | 0.2245 | No |
Table: GSEA details [plain text format]

  

Fig 2: GOBP\_PHAGOSOME\_ACIDIFICATION      
 Blue-Pink O' Gram in the Space of the Analyzed GeneSet

  

Fig 3: GOBP\_PHAGOSOME\_ACIDIFICATION: Random ES distribution      
 Gene set null distribution of ES for **GOBP\_PHAGOSOME\_ACIDIFICATION**
